# CeRNA plays a key role in the induction of cardiovascular diseases by environmental endocrine disruptor exposure

**DOI:** 10.1265/ehpm.25-00165

**Published:** 2026-02-27

**Authors:** Yingxi Zeng, Jie Xu, Jie Yu

**Affiliations:** School of Public Health, Zunyi Medical University, Zunyi, Guizhou, 563000, P. R. of China

**Keywords:** CeRNA, mRNA, miRNA, Environmental endocrine disruptors, Cardiovascular disease

## Abstract

**Background:**

Competing endogenous RNAs (ceRNAs) represent a novel mechanism involving interactions among different RNAs, playing a crucial role in the gene regulatory networks throughout the life cycle. CeRNAs are implicated in cardiovascular diseases (CVDs) caused by environmental endocrine disruptors (EDCs); however, existing studies are not yet systematic, and the mechanisms underlying their effects remain unclear.

**Objective:**

This study aimed to systematically elucidate the role of ceRNAs in EDC-induced CVDs and provide valuable insights regarding disease mechanisms and developing new therapeutic strategies.

**Methods:**

Comprehensive searches for research related to EDC-induced cardiovascular diseases were conducted across PubMed, Web of Science, and ScienceDirect databases. Eligible studies were screened, and those containing information on the regulatory mechanisms of ceRNAs were extracted and analyzed.

**Results:**

Notably, ceRNA-mediated effects of EDC exposure on CVDs mainly occurred through four pathways. First, upon exposure to EDCs, micro RNAs, messenger RNAs (mRNAs), long-chain non-coding RNAs, circular RNAs are differentially regulated, activating signaling pathways such as nuclear factor erythroid 2-related factor 2 and p38 mitogen-activated protein kinase/nuclear factor-κB, which lead to atherosclerosis. Second, EDC exposure alters mRNAs and proteins involved in ceRNA networks, activating the PTEN-induced kinase 1/Parkin and transforming growth factor-β1/LIM domain kinase 1 signaling pathways, leading to cardiomyopathy. Third, EDCs increase ceRNA-related mRNA levels, thereby raising the risk of CVDs. Lastly, ceRNAs participate in EDC exposure to upregulate nitric oxide or reactive oxygen species, ultimately causing vascular diseases.

**Conclusion:**

Altogether, the findings of this study show that ceRNAs hold significant potential for identifying target genes and signaling pathways associated with CVDs, which may facilitate deeper studies into CVD management.

**Supplementary information:**

The online version contains supplementary material available at https://doi.org/10.1265/ehpm.25-00165.

## Graphical Abstract

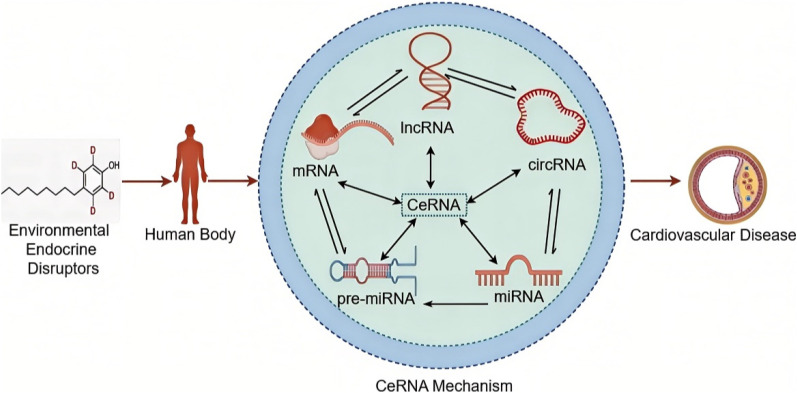

## 1. Introduction

Cardiovascular disease (CVD) is the leading cause of morbidity and mortality worldwide, with epidemiological studies indicating that approximately 17.9 million CVD-related mortalities per year, accounting for 32% of all global deaths. The World Health Organization projects further increases in CVD mortality by 2030 of up to 24 million, with a mortality rate as high as 40%. Notably, CVD places first in the composition ratio of urban and rural populations regarding disease-related deaths in China, with its prevalence rate continuously increasing. This makes CVD a substantial threat to public health and places a heavy economic burden on residents and society, making it the current world’s “number one killer” [1, 2].

Beyond established risk factors such as obesity [3, 4], diabetes mellitus [5, 6], hypertension [7, 8], high blood cholesterol [9, 10], smoking [11, 12], sex [13, 14], and age [15, 16], which are directly linked to CVD pathogenesis, environmental endocrine disruptors (EDCs) also contribute to CVD development. EDCs are exogenous chemicals released into the environment through industrial and daily living activities of humans, and they can interfere with normal hormone levels in humans and animals [17, 18]. Recent studies show that EDCs trigger endocrine disruption [19], leading to hormonal dysregulation, developmental and reproductive abnormalities [20–22], organ pathologies [23], and increased teratogenesis risks [24]. Evidence from animal models, clinical observations, and epidemiology further highlights the effects of EDCs on the reproductive system [25, 26], cancer [27], metabolism [28], obesity [29], neuroendocrine system [30, 31], and CVDs [32]. Although research confirms that EDC exposure induces CVD, its full impact remains unelucidated.

Competing endogenous RNAs (ceRNAs) represent a novel mechanism that involves interactions between different RNA molecules, including protein-coding messenger RNAs (mRNAs), long-chain non-coding RNAs (lncRNAs), pseudogenes, and circular RNAs (circRNAs). These RNAs are widely involved in the development-related physiological processes, such as differentiation and metabolism [33, 34], and play vital roles in the gene regulatory networks across the life cycle. Furthermore, microRNAs (miRNAs) have been shown to no longer be regulated solely by binding to their target gene mRNA, but they can compete for binding with other non-coding RNAs (ncRNAs), such as lncRNA and circRNA, thereby affecting the regulation of target genes [35–37]. CeRNAs have been implicated in EDC-induced CVDs, yet related studies lack a systematic approach. Key limitations include the following three aspects: (1) Fragmented research focusing on individual EDCs, specific ceRNA pairs, or isolated CVD outcomes without a holistic network perspective; (2) Predominantly observational findings without robust in vivo or in vitro validation of causality; and (3) Inconsistent methodologies that hinder cross-study comparisons and broad conclusions. Building on the existing literature, this study aimed to investigate and summarize the regulatory roles of ceRNAs in EDC exposure-induced CVDs and their related mechanisms. Additionally, the study discusses the prospects of ceRNAs, offering critical insights into disease pathogenesis.

## 2. Methods

### 2.1 Keyword search

A comprehensive search was conducted across three scientific databases, namely PubMed, Web of Science, and ScienceDirect. The following keywords were employed: “Environmental endocrine disruptors,” “environmental hormones,” “endocrine active compounds,” “endocrine disruptors,” “edcs” OR “EED,” “EEDS,” “endocrine disrupting chemicals,” “endocrine active compounds,” “edc,” “nonylphenol,” “phthalates,” “polychlorinated biphenyl,” “organo-chlorine pesticide,” “octyl phenol,” “cardiovascular diseases,” “circulatory system diseases,” “hypertension,” “blood Pressure,” “myocardial infarction,” “MI,” “ischemic heart disease,” “IHD,” “coronary heart disease,” “pulmonary heart disease,” “peripheral arterial disease,” “PAD,” “stroke,” and “macrovascular diseases.” These terms were combined using Boolean operators “AND”, “OR” as detailed in Supplementary Material Table S1. The search was restricted to studies published between January 1, 2006, and January 1, 2026, from which literature relevant to ceRNA was extracted. Two independent researchers performed the search and screening of articles in accordance with the PRISMA guidelines, and the study protocol is registered with Prospero https://www.crd.york.ac.uk/PROSPERO/recorddashboard, CRD420261281731.

### 2.2 Inclusion and exclusion criteria

Inclusion Criteria: 1) Experimental study design (e.g., clinical trials, field trials, animal experiments, cell experiments); 2) Concurrent reporting of endocrine disrupting chemicals (EDCs), cRNA, and cardiovascular disease (CVD); 3) Study outcomes involving CVD-related diseases.

Exclusion Criteria: 1) Studies with other research designs, such as observational studies; 2) Reviews, meta-analyses, guidelines, conference abstracts, paper abstracts, case reports, etc.; 3) Studies published before 2006; 4) Studies lacking concurrent information on EDCs, CVD, and ceRNA; 5) Non-English studies.

### 2.3 Literature selection

**Fig. 1:**
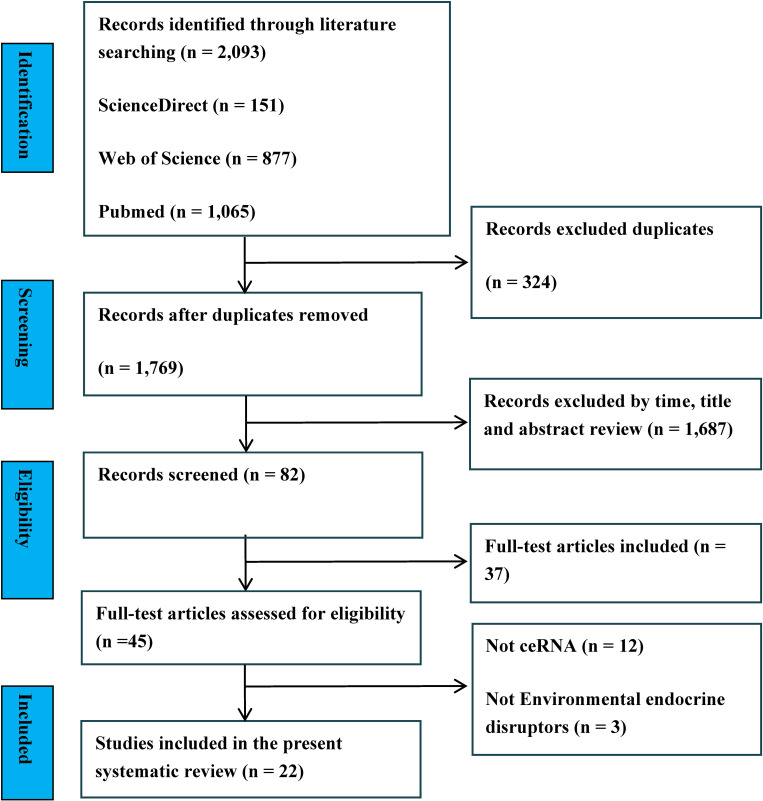
Literature search flowchart.

Two researchers (Yingxi Zeng and Jie Xu) independently searched three databases (PubMed, ScienceDirect, and Web of Science) and imported a total of 2,093 articles into EndNote X9 software. Using the software’s automatic duplication detection feature, 324 articles duplicated across different databases were removed. After applying time restrictions and carefully reviewing article titles and abstracts, 1,687 articles were excluded. After reviewing full texts, 37 were excluded. Further examination of the remaining 45 articles revealed that 12 did not include ceRNA in their results, and 3 were not endocrine disruptors. Ultimately, 22 articles were included. Any disagreements during the screening process were resolved through discussion until consensus was reached. Otherwise, a third researcher (Jie Xu) made the final decision. The study selection procedure is detailed in the PRISMA flow diagram in Fig. 1.

## 3. Results

CVD-associated EDCs are primarily classified into the following four structural categories: halogen- and sulfur-free compounds, halogenated compounds, pyrethroid compounds, and heavy metals. To date, at least 12 EDCs have been identified across these four categories, including bisphenol A (BPA) and its analogs, nonylphenol (NP), perfluoroalkyl substances, 2,3,7,8-tetrachlorodibenzo-p-dioxin (TCDD), cadmium, perfluorooctanesulfonic acid, polychlorinated biphenyls (PCBs), dibutyl phthalate, other phthalates, soy isoflavones, and octabromodiphenyl ether mixtures.

CeRNA networks implicated in EDC-induced CVDs involve multiple RNA types, including mRNAs, miRNAs, lncRNAs, circRNAs, small nucleolar RNAs (snoRNAs), non-coding RNAs (ncRNAs), and small nuclear RNAs (snRNAs). Additionally, the key signaling pathways include the nuclear factor erythroid 2-related factor 2 (Nrf2) pathway, p38 mitogen-activated protein kinase (MAPK)/nuclear factor (NF)-κB axis, PTEN-induced kinase 1 (PINK1)/Parkin-mediated mitophagy, and transforming growth factor (TGF)-β1/LIM domain kinase 1 (LIMK1) cascade. A systematic overview of relevant genes, proteins, and pathway components is presented in Table 1.

**Table 1:** Partial list of information on ceRNA involvement in environmentally induced endocrine disruptors and cardiovascular disease.

**No.**	**Environmental endocrine ** **disruptors**	**Abbreviation**	**Type of experiment**	**Models**	**CeRNA network**	**Reference**
1	2,3,7,8-Tetrachlorodibenzo-p-dioxinand Polychlorinated Biphenyls	TCDD, PCBs	*In vivo*	ApoE mice	MiRNA, miRNA-mRNA, miRNA-26a-5p, miRNA-193a-3p, 1312mRNA, miRNA-30c-5p, miRNA-130a-3p, miRNA-376a-3, Mvd, Lss Hmgcr, Sqle, mRNA, proteins 2	(Shanet, et al., 2020)
2	Bisphenol A	BPA	*In vivo*	Male CD1 mice	CYP11B2mRNA	(Marmugi, et al., 2014)
3	Perfluoroalkyl substances and pentadecafluorooctanoic acid and perfluorooctanesulfonic acid	PFAS, PFOA, PFOS	*In vitro*	Carcinoma cells HAC15	PCG-1, mRNA	(Caroccia, et al., 2023)
4	Bisphenol A	BPA	*In vivo*	Male mice	MRNA, ERβ, Cyp19a1, PhAKT^ser473^, PhGSK-3β^ser9^, PhAR^ser308^, TNF-a, IL-6, IL-8 mRNA, TxR1, p38 MAPK/NF-kB pathway	(Jiang, et al., 2015)
5	Cadmium	Cd	*In vitro*	Human Cells	MRNA	(Fittipaldi, et al., 2019)
6	2,3,7,8-tetrachlorodibenzo-p-dioxin	TCDD	*In vivo*	C57BL/6 mice	MRNA, Nqol, Icaml, PAI1	(Brulpor, et al., 2017)
7	Polychlorinated biphenyl 126 and Perfluorooctane sulfonic acid	PCB126, PFOS	*In vivo*	Male C57BL/6 mice	MRNA, ncRNA, IncRNA, miRNA, snoRNA, snRNA	(Deng, et al., 2020)
8	Bisphenol A	BPA	*In vivo*	Female lambs	MRNA, ncRNA, IncRNA, miRNA, snoRNA, snRNA	(Dou, et al., 2023)
9	Bisphenol A	BPA	*In vivo*	Female sheep	MRNA, AP-1, EGR1, EGFR	(Koneva, et al., 2017)
10	Dibutyl phthalate	DBP	*In vivo*	Human endothelial cell	ERK1/2, PI3K-Akt, GPER	(Kokai, et al., 2022)
11	Nonylphenol	NP	*In vivo and * *in vitro*	Pregnant rats and H9C2 cells	PGC-1a, NRF-1, SIRT1, PGC-1a, NRF-1, TFAM, PGC-1a/NRF-1/TFAM pathway	(Ni, et al., 2023)
12	Polychlorinated biphenyls	PCBs	*In vivo and * *in vitro*	Human cells and mice	LncRNA HDAC7-AS1, MIR-7-5p, argonaute 2, PCB29-pQ, mRNA, TGF-β2, MIR-7-5p, HDAC7-AS1, HDAC7-AS1	(Yang, et al., 2022)
13	Genistein	/	*In vivo and * *in vitro*	H9C2 cells and ICR mice	MiR-451, TIMP2	(Gan, et al., 2019)
14	Bibutyl phthalate	DBP	*In vivo and * *in vitro*	Sprague-Dawley rats and human cell	MRNA, TGFB1I1, β1, AR	(Wu, et al., 2024)
15	Bisphenol A	BPA	*In vivo*	Patients with T2DM	MRNA, GLB1, p16, p21, p53, ERRγ	(Soundararajan, et al., 2019)
16	Bisphenol AF	BPAF	*In vivo and * *in vitro*	Zebrafish and human cardiac Myocytes	MRNA	(Gu, et al., 2020)
17	Octabromodiphenyl ether mixture, DE-79	BDEs-DE-79	*In vivo*	Wistar rats	MRNA, nNOS-IR, NO, AVPergic	(Alvarez- Gonzalez, et al., 2020)
18	Di(2-ethylhexyl)phthalate	DEHP	*In vivo and * *in vitro*	ApoE mice and foam cell	CeRNA, miR-145-5p, GAS5, IncRNA, GAS	(Liu, et al., 2022)
19	Polychlorinated biphenyl 77	PCB77	*In vivo*	Male apolipoprotein ApoE mice	MRNA, ATlaR	(Arsenescu, et al., 2011)
20	Phthalate	PAEs	*In vivo*	Menreceiving clinical infertility care	MiRNA, ncRNA, tRFs, piRNAs, MAPK1, BMPR1A/2, PTEN, TGFBR2, TP53, APP	(Oluwayiose, et al., 2023)
21	Phthalate	PAEs	*In vivo*	Crowd	miRNA-155, miRNA-208a	(Moawad et al., 2024)
22	Endosulfan	/	*In vivo and * *in vitro*	mice and HUVECs	miR-140-5p, pmiR-KCNQ1OT1-WT/MUT, miR-137-3p, miR-NC	(Zhang et al., 2025)

### 3.1 Role of miRNAs and lncRNAs in EDC-induced atherosclerosis (AS)

AS is the leading cause of CVD [38], and EDCs have been reported as inducers of AS [39]. In the ceRNA network, miRNAs, a class of short-stranded RNA (length: approximately 22 nt), regulate gene expression [40]. In humans, co-exposure to TCDD and PCBs can result in alterations in 68 miRNAs and 1,312 mRNAs, with miR-26a-5p, miR-193a-3p, and miR-30c-5p involved in a network associated with the specific TCDD–PCB co-response. These networks are further associated with the developmental and functional frameworks of the cardiovascular system. Additionally, miR-130a-3p and miR-376a-3p have been identified as novel players in the regulation of TCDD/PCB-induced AS pathways [41]. Diethylhexylphenyl dicarboxylate (DEHP) can promote lipid uptake by downregulating lncRNA growth arrest-specific 5 (GAS5) and altering miR-145-5p regulation, thereby accelerating AS onset in mice and altering their lipid profiles. In contrast, in human umbilical vein endothelial cells (HUVECs) and vascular smooth muscle cells (VSMCs), DEHP upregulates GAS5 and downregulates miR-145-5p, with GAS5 acting as a ceRNA for miR-145-5p to regulate VSMC proliferation, apoptosis. Notably, the GAS5 expression correlates with that of miRNA-145-5p, and GAS5 knockdown can reverse the effects of DEHP on foam cell formation and oxidized low-density lipoprotein uptake [42].

**Fig. 2:**
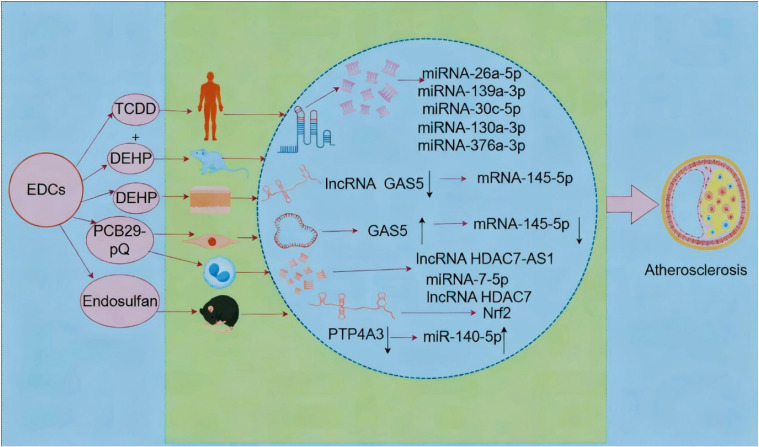
Mechanism diagram of the role of EDCs in the induction of atherosclerosis via miRNA and lncRNA.

Cadmium chloride exposure in HUVECs can significantly increase estrogen receptor-β and cytochrome P450 family 19 subfamily A member 1 expression at mRNA/protein levels, with taxol resistance protein 1 overexpression associating with p38 MAPK/NF-κB pathway activation, causing endothelial cell (EC) injury and vascular dysfunction, which further leads to AS [43]. Exposure of HUVECs to 2,3,5-trichloro-6-phenyl-[1,4]-benzene (PCB29-pQ) results in binding of lncRNA histone deacetylase 7 antisense RNA 1 (HDAC7-AS1) to miR-7-5p via Argonaute 2. Reportedly, HDAC7-AS1 sponges miR-7-5p, inhibiting TGF-β2 from binding miR-7-5p, and thereby promoting endothelial damage, vascular inflammation, and plaque formation to accelerate AS [44]. Additionally, PCB29-pQ can drive macrophage/monocyte polarization to cluster of differentiation (CD)163^+^ macrophages via the Nrf2 signaling pathway to promote AS development [45]. Notably, PCB29-pQ exposure has been shown to lead to lipid accumulation, endoplasmic reticulum stress response, apoptosis, and pro-inflammatory cytokine release via CD36, ultimately contributing to AS [46]. MiR-140-5p targets PTP4A3 and KCNQ1OT1, inhibiting endothelial cell migration and MAPK/ERK/PI3K/AKT pathways. Its downregulation by Endosulfan promotes migration, a mechanism also observed in atherosclerotic mice [95]. Overall, these findings show that long-term EDC exposure differentially regulates ceRNAs (miRNAs, mRNAs, and lncRNAs) or activates Nrf2 and p38 MAPK/NF-κB pathways to induce AS and CVD (Fig. 2).

### 3.2 Role of ceRNA in heart disease caused by exposure to EDCs

#### 3.2.1 MRNA mediates the development of cardiomyopathy by BPA and its substitutes

Owing to health risks posed by BPA, various alternatives have been developed, though many also exhibit toxicity to the human body. Reportedly, exposure to BPAF, a primary alternative to BPA, suppresses mRNA expression of key genes involved in cardiac development, induces ROS production, antioxidant enzyme inhibition, mitochondrial dysfunction, and oxidative DNA damage. It has also been shown to reduce cardiomyocytes and endocardial cell populations in the heart, along with cardiac reduction in a transgenic zebrafish model [47]. Even at low concentrations, BPA can trigger cardiac developmental defects, significantly reducing beating rates and prolonging contraction/relaxation times in human induced pluripotent stem cell-derived cardiomyocytes (hiPSC-CMs) dose-dependently. Quantitative proteomics has revealed elevated basement membrane components, including collagen type IV alpha (COL4A)1, COL4A2, laminin subunit gamma 1, and nidogen 2, along with increased troponin C1 and SERPINE1 mRNA binding protein 1 levels in BPA-treated hiPSC-CMs, with network analysis indicating altered extracellular matrix remodeling and links to cardiac disease-related genes [48].

In rat offspring exposed to BPA, 17α-ethinylestradiol (EE), or carboxymethylcellulose, males showed weight loss, whereas EE-exposed females exhibited increased heart weights. Conversely, collagen accumulation was increased in males, whereas collagen reduction was observed in the hearts of BPA- or EE-exposed females. In contrast, myocardial degeneration occurred in both male and female rats [49]. Notably, cardiomyocyte Ca^2+^ transport regulation has been critically implicated in cardiac pathophysiology, and reduced sarcoplasmic reticulum Ca^2+^ load and inhibited L-type Ca^2+^ channels (which can alter Ca^2+^ transport-regulating proteins) have been shown to mediate the effects of BPAF on myocyte contraction and Ca^2+^ transport regulation. Reportedly, BPAF exposure for 4–5 d does not result in hypertrophy of normal hiPSC CMs, although it can significantly exacerbate the endothelin-1-induced cardiomyocyte hypertrophic phenotype, characterized by increased cell size and overexpression of the hypertrophic marker proBNP. These findings indicate that chronic exposure to BPA and some of its analogs may lead to contractile dysfunction and abnormal Ca^2+^ transport regulation, making it a risk factor for cardiac hypertrophy in humans [50].

**Fig. 3:**
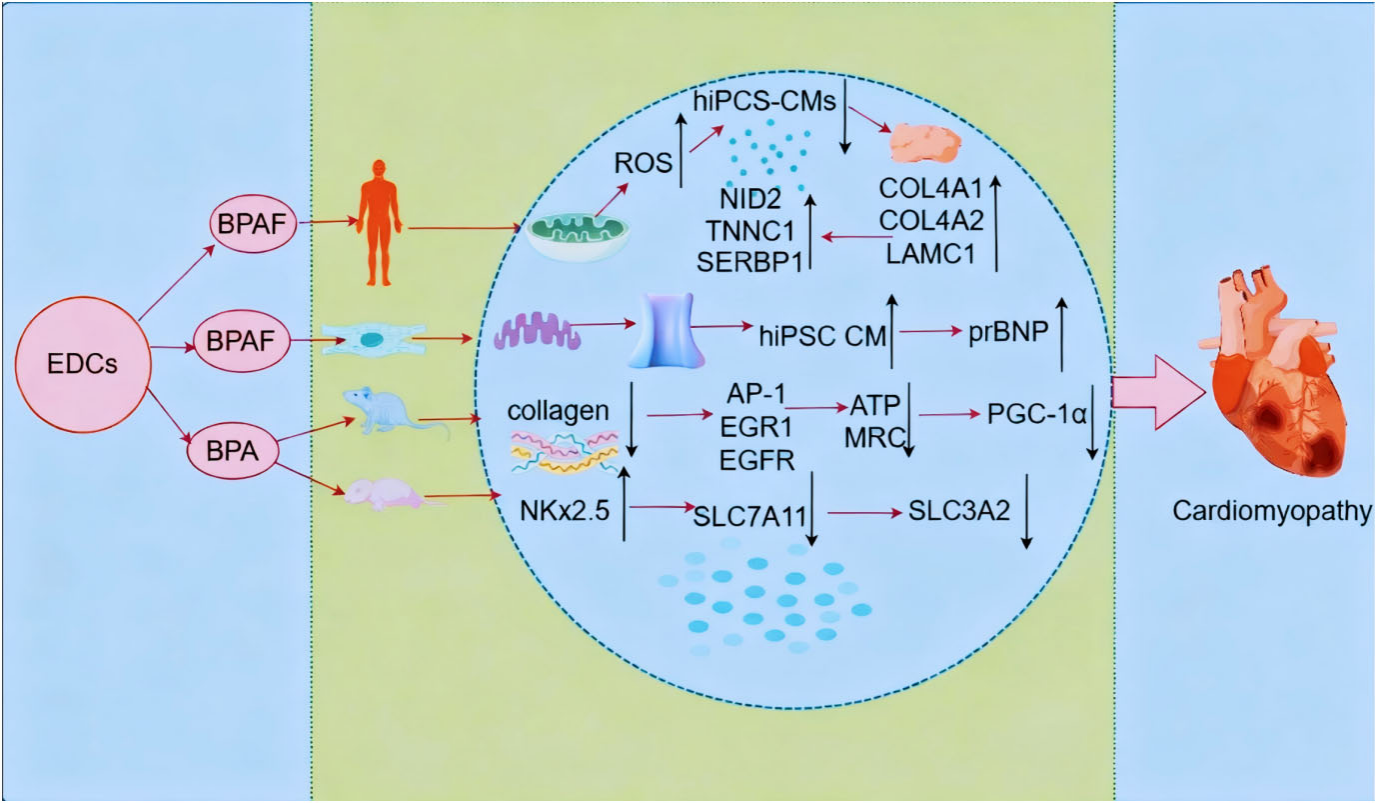
Schematic representation of the mechanism of ceRNA involvement in EDC-induced cardiomyopathy.

A study reported that prenatal exposure to BPA and postnatal overfeeding induced transcriptional changes in 85 significantly differentially expressed genes, underscoring the association of BPA with obesity, hypertension, and heart disease, with activator protein-1, early growth response protein 1, and epidermal growth factor receptor being the key BPA-affected hub proteins [51]. Additionally, significantly reduced ATP production, mitochondrial membrane potential (Ψm) dissipation, and mitochondrial respiratory complex activity observed in cardiomyocytes of BPA-exposed rats were found to further downregulate peroxisome proliferator-activated receptor gamma coactivator (PGC)-1α expression and induce its hypermethylation in rat heart tissues [52]. Changes in the fetal heart structure have been observed in BPA-treated mice. An in vivo study reported an increase in NK2 homology box 2.5 following ferroptosis induction, suggesting that BPA induces developmental abnormalities in the fetal heart. Furthermore, solute carrier family 7 member 11 (SLC7A11) and solute carrier family 3 member 2 (SLC3A2) downregulation in the low- and high-dose groups has been correlated with BPA-induced developmental abnormalities in the fetal heart via System Xc-mediated inhibition of glutathione peroxidase 4 (GPX4) expression. Findings in AC-16 cells have confirmed that BPA exposure at various concentrations significantly decreases cell viability and suppresses GPX4 expression by inhibiting System Xc (SLC3A2 and SLC7A11 downregulation) [53]. These findings show that ceRNA mediates the promotion of cardiomyopathy through BPA and its analogs (Fig. 3).

#### 3.2.2 mRNA-mediated myocardial fibrosis from NP exposure

**Fig. 4:**
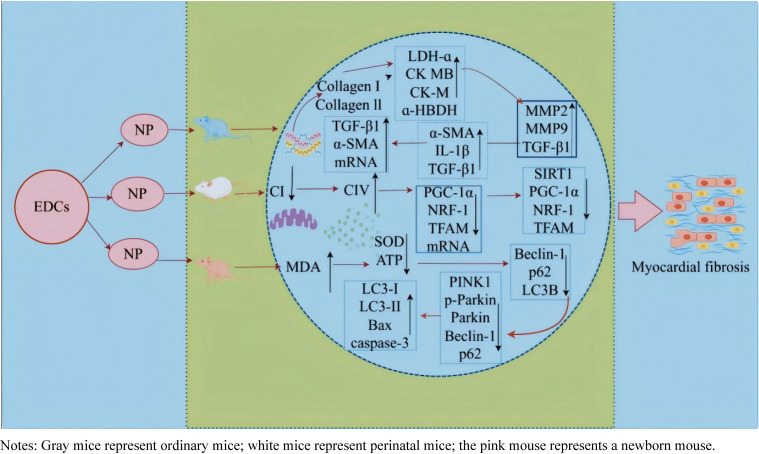
Mechanism diagram showcasing CeRNA involvement in endocrine disruptor-induced myocardial fibrosis.

In recent years, NP exposure in mice has been shown to induce myocardial fibrosis, with both collagen I and III levels increasing with higher NP doses. Reportedly, serum myocardial enzymes, including aspartate aminotransferase, creatine kinase, creatine kinase isoenzyme, lactate dehydrogenase, and α-hydroxybutyrate dehydrogenase, are significantly elevated. NP exposure also causes damage to the left ventricular anterior wall, making it thinner, while the left ventricular end-diastolic diameter increases [54]. A detailed study revealed that NP exposure upregulates the expression of key myofibrosis-associated proteins, including collagen I and IIII, matrix metallopeptidase (MMP)2, MMP9, TGF-β1, α-smooth muscle actin (SMA), and interleukin (IL)-1β, and TGF-β1, and genes encoding collagen I collagen III, TGF-β1, and α-SMA mRNA. These findings suggest activation of the TGF-β1/LIMK1 signaling pathway following NP exposure [55]. Notably, perinatal NP exposure has been shown to cause mitochondrial damage in mouse offspring, characterized by endolysis with vacuolization and decreased Ψm. It also reduced the activity of mitochondrial respiratory enzyme complex I while increasing complex IV activity. Furthermore, mRNA and protein levels of cardiac mitochondrial biogenesis regulators, including PGC-1α, nuclear respiratory factor (NRF)-1, and transcription factor A, mitochondrial (TFAM), were decreased. Similarly, in H9C2 cells exposed to NP, Ψm was significantly reduced, along with protein expression of sirtuin1, PGC-1α, NRF-1, and TFAM [56]. NP exposure has been shown to increase malondialdehyde (MDA) levels while decreasing superoxide dismutase (SOD) activity and ATP content in myocardial tissues. Reportedly, the mRNA levels of autophagy-related genes, including Beclin-1, p62, and microtubule-associated protein 1 light chain 3 (LC3)B, are increased. Correspondingly, mitochondrial autophagy-related proteins (such as PINK1, phosphorylated [p]-Parkin, Parkin, Beclin-1, p62, LC3-I, LC3-II, and the LC3-II/I ratio) and apoptosis-related proteins (B-cell lymphoma [Bcl]2-associated X protein and caspase-3) were upregulated, and the mitochondrial membrane protein translocase of the outer mitochondrial membrane 20 and the anti-apoptotic protein Bcl-2 were downregulated [57]. Overall, NP exposure induces changes in mRNA and protein expression, activating signaling pathways such as TGF-β1/LIMK1 and PINK1/Parkin, which contribute to the development of myocardial fibrosis (Fig. 4).

#### 3.2.3 MiRNA-mediated EDCs influence cardiac lesions

Isoprenaline-induced cardiac hypertrophy in mice treated with genistein flavonoids has shown that genistein flavonoids downregulate miR-451 expression while upregulating its target gene tissue inhibitor of metalloproteinase 2 [58]. Similarly, another study demonstrates that miR-451 plays a crucial role in genistein-mediated improvement of isoprenaline-induced cardiac hypertrophy both in vitro and in vivo [59]. Reportedly, chronic exposure of juvenile zebrafish hearts to low doses of iodoxynitrile and diethylstilbestrol (DES) has been shown to result in ventricular deformation and volume increase. DES exposure specifically causes altered ventricular morphology and upregulates endothelium-related genes involved in vascular homeostasis, including *angptl1b*, *mhc1lia*, *mybpc2a*, *ptgir*, *notch1b*, and *vwf* [60].

**Fig. 5:**
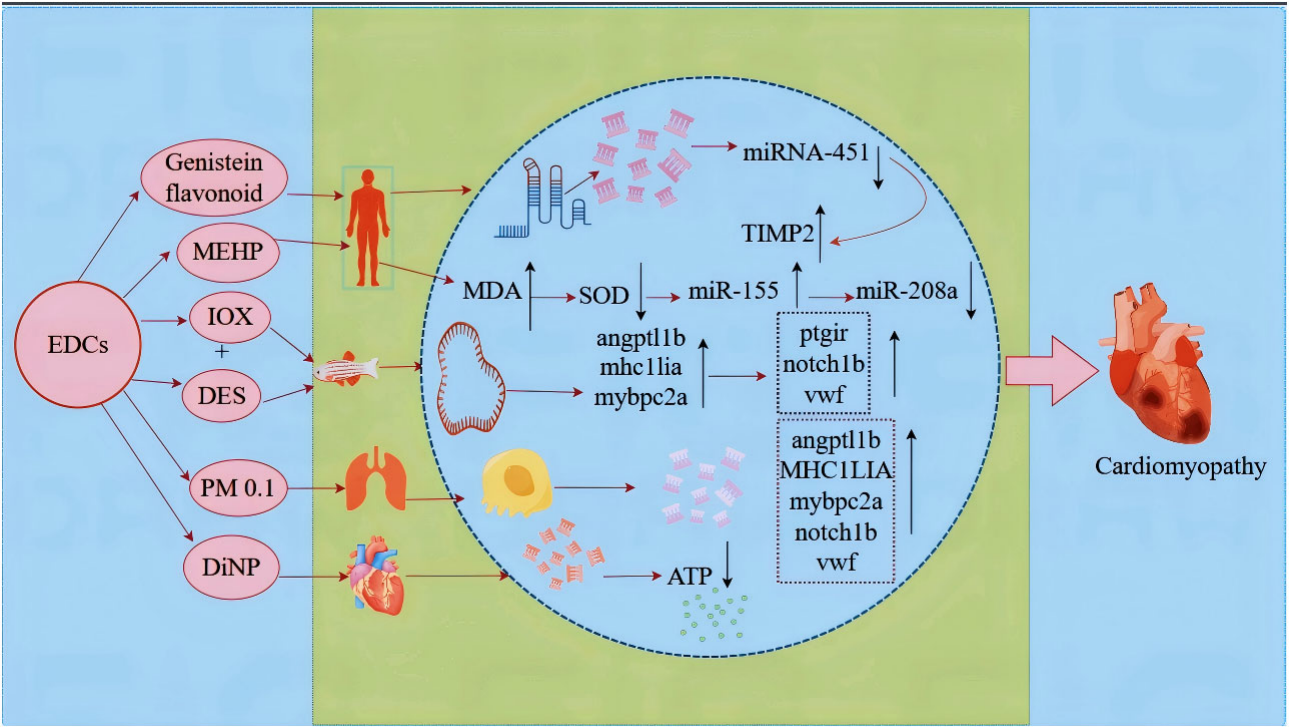
CeRNA mediates the mechanism through which BPA and its surrogates promote cardiac lesions.

Notably, smaller particulate matter (PM 0.1) can penetrate the alveolar system, disperse, and accumulate in cells, thereby impacting the cardiovascular system [61]. Reportedly, diisononyl phthalate (DiNP) interferes with cardiac energy-transducing enzymes, leading to insufficient ATP production by cardiac cells for their morphological and physiological functions. Because the heart requires a continuous ATP supply to support contraction, relaxation, and prevent cardiomyopathy, DiNP exposure indirectly contributes to the development of cardiomyopathy [62]. In a study employing the zebrafish model, DES exposure induced upregulation of endothelial-related genes that maintain vascular homeostasis, such as *angptl1b*, the *MHC1LIA* gene family, *mybpc2a*, *notch1b*, and *vwf*, thereby affecting cardiac function [63]. Campinho MA reported that both ioxynil and DES increased the risk of cardiovascular and thyroid dysfunction in zebrafish [64]. A study found that phthalate exposure (via urinary MEHP) is linked to coronary heart disease. Altered oxidative stress and elevated levels of miR-155 and miR-208a, which correlate with MEHP, may be key mechanisms [96] (Fig. 5).

### 3.3 Exposure to EDCs leads to increased CVD risk at the mRNA level

TCDD is involved in adipose tissue and liver metabolism, inflammation, xenobiotic metabolism, and endocrine disruption, and its mRNA levels are known to be differentially regulated in males and females [65]. In a case-control study, exposure to BPA substitutes altered the osteoprotegerin gene and mRNA expression in a population, suggesting that elevated blood and urine BPA levels may contribute to the severity of vascular complications in patients with type 2 diabetes mellitus (T2DM) through vascular calcification [66]. Furthermore, repeated exposure to low-dose BPA has been shown to upregulate various markers, including N-calmodulin, waveform protein, CD44, and αα-SMA, with a decrease in the levels of E-calmodulin in MCF-7 cells. The BPA-induced mitochondrial biogenesis met the bioenergetic demands of MCF-7 cells through PGC-1α/NRF1/estrogen-related receptor (ERR)α signaling requirements, favoring metastatic attack, and PGC-1α knockdown inhibited mitochondrial biogenesis and epithelial–mesenchymal transition (EMT) in BPA-exposed MCF-7 cells [67]. Reportedly, hepatic mRNA levels of *Nqo1*, *Icam1*, and *PAI1* are significantly increased in mice exposed to PCB126 and perfluorooctane sulfonate. Moreover, plasma levels of markers of fibrosis and thrombosis (namely *TGFB*–*PAI1*) were also significantly elevated due to inhibition of the cytoplasm-to-nucleus translocation of the transcription factor AR, and liver damage was observed in the mixture-exposed group. These results highlighted an increased risk of CVD [68]. In another study, mice injected with angiotensin (Ang)II containing PCB77 exhibited increased systolic blood pressure, and nearby adipose tissue showed increased mRNA abundance of pro-inflammatory cytokines, along with elevated expression of Ang and Ang type 1a receptor of the renin-angiotensin system [69].

**Fig. 6:**
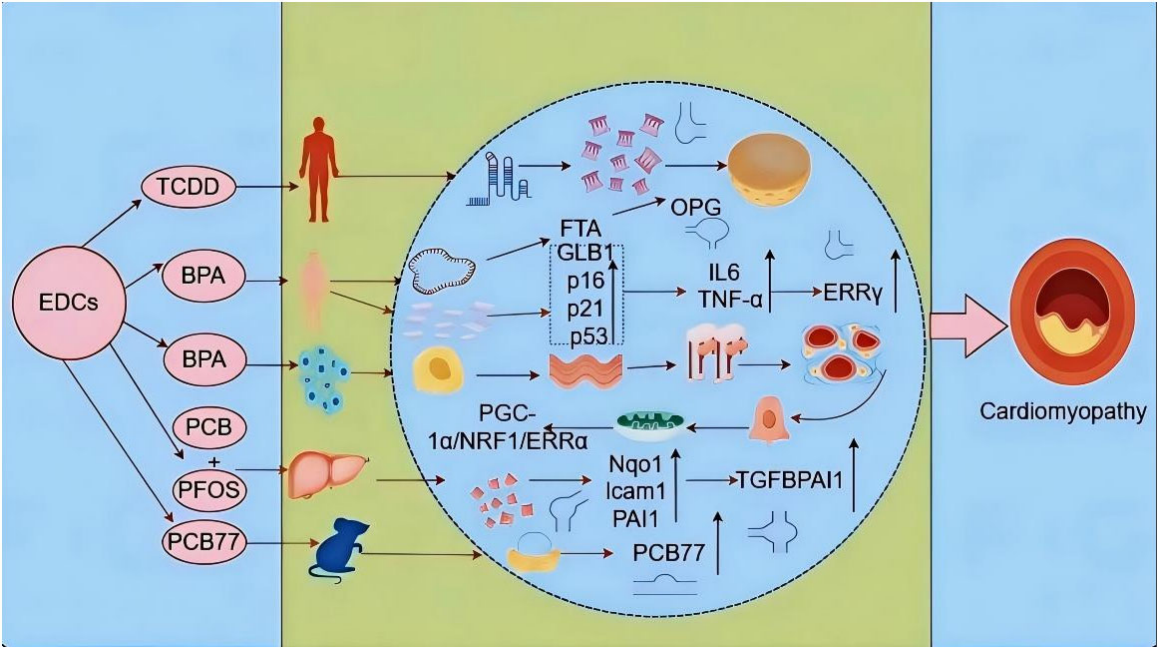
Exposure to EDCs increases the risk of cardiovascular disease at the mRNA level.

Epidemiological evidence suggests the association of T2DM with an increased mortality risk of CVD across race and sex, along with being the most common complication of CVD [70, 71]. Notably, measurement of systemic BPA levels revealed significantly higher serum BPA levels in patients with T2DM compared with those in individuals with normal glucose tolerance, positively correlating with poor glycemic control and insulin resistance. Furthermore, patients with T2DM had significantly elevated mRNA levels of markers of senescence (galactosidase beta 1, p16, p21, and p53) and inflammation (IL-6 and TNF-α), telomere shortening, and elevated levels of ERRγ [72] (Fig. 6).

### 3.4 CeRNA-mediated alteration of nitric oxide (NO) by exposure to EDCs induces vascular disease

RNA sequencing analysis of dibutylphthalate (DBP)-treated HUVEC reveals that TGF-β1-induced overexpression of transcript 1 promotes vessel formation and migration, with acute DBP exposure increasing endothelial tube formation and NO production in the human EC line EA.hy926 [73]. Reportedly, DBP mediates phosphorylation of extracellular signal-regulated kinase (ERK)1/2, protein kinase B (Akt), and endothelial NO synthase (eNOS) to increase NO production [74]. In a study, following perinatal PentaBDE mixture (DE-79) exposure, AVP-IR mRNA expression and systemic release were more pronounced in response to hyperosmotic stimuli. Notably, neuronal NO synthase-IR and mRNA expression were affected in the same nuclei, suggesting that NO dysregulation may lead to disruption of the AVPergic system [75]. In contrast, another study reported that BPS exposure in male mice decreased NO and increased ROS production in HUVECs and led to Ψm loss, ultimately resulting in vascular damage in mice [76]. These findings suggest that different ceRNAs or signaling pathways exhibit differential involvement and detection approaches.

Tetrabromobisphenol A (TBBPA), one of the most widely used brominated phenolic compounds, is used as a flame retardant in plastics, textiles, and paper worldwide [77, 78]. A study found that TBBPA altered 5-hydroxytryptamine (5-HT), histamine, and KCl contraction by interfering with 5-HT and histamine receptors, involving Ca^2+^-regulated L-type Ca^2+^ and BK calcium 1.1 alpha and beta1 subunit channels, along with soluble guanylate cyclase (sGC) and cyclic guanosine phosphorothioate-dependent protein kinase (PKG). This effect was attributed to the association of TBBPA with the NO/sGC/cyclic guanosine monophosphate (cGMP)/PKG pathway and interference with calcium inward currents, which impaired the major vasodilator mechanism in human umbilical arteries [79].

**Fig. 7:**
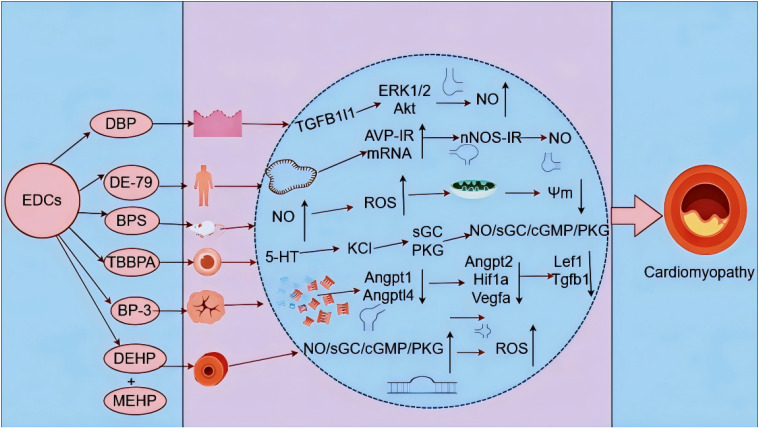
CeRNA mediates NO alteration via EDC exposure to induce vascular disease.

Benzophenone-3 (BP-3) is an endocrine disruptor and a common ingredient in sunscreens and other personal care products [80]. Reportedly, BP-3 downregulated *Angpt1*, *Angptl4*, *Angpt2*, *Hif1a*, *Vegfa*, *Lef1*, *Tgfb1*, *Timp1*, *Twist1*, and *Fn1* in low-fat diet tumors, which stabilized the vasculature in epithelial cancers, inhibited EMT, and suppressed spindle cell tumors [81]. Additionally, both PCB153 and PCB77 bound to estrogen receptor (ER) alpha, thereby inducing agonistic or antagonistic responses, respectively, to positively or negatively regulate angiogenic processes and behave like estrogens in endothelial cells [82]. Application of ER and G protein-coupled ER inhibitors ICI182, 780, and G-15 has been shown to reverse DBP-mediated phosphorylation of ERK1/2, Akt, and eNOS, along with increased NO production [83]. Reportedly, short-term exposure to DEHP interferes with serotonin and histamine receptors, whereas its long-term exposure exhibits the same vasorelaxant mechanism as estrogen, interfering with L-type Ca^2+^ channels via the NO/sGC/cGMP/PKG signaling pathway [84]. Moreover, DEHP and MEHP exposure can lead to testicular damage and accelerate cellular senescence through ROS accumulation. RNA sequencing analyses have revealed activation of Forkhead box O (FOXO) signaling and longevity regulatory pathways in response to excessive ROS accumulation, along with that of associated genes and proteins. Constructing the ceRNA network has shown that it plays a role in regulating FOXO signaling and longevity pathways in response to ROS accumulation and cellular senescence [85]. Overall, these studies confirm the involvement of ceRNAs in the overexpression of NO or ROS via EDCs to induce vascular disease (Fig. 7).

## 4. Summary and prospects

EDCs are widely present in daily lives and environments and exhibit great potential to harm human and animal health [86]. Evidence from both observational and experimental studies highlights the adverse effects of EDCs in humans, including chronic autoimmune diseases [87], skeletal abnormalities, female infertility, reproductive disorders [88], neuroscience [89], metabolic disorders [90, 91], and CVDs [92, 93]. Notably, CVDs are the leading cause of mortality worldwide, with EDCs being a major contributing factor. Although it is generally known that EDCs exert adverse effects on health, the continuous development of science and technology has made it challenging to avoid them. Therefore, elucidating the mechanism of CVDs caused by EDCs may provide ideas for addressing such challenges.

This study demonstrates the key role of ceRNAs in EDC-mediated induction of CVD. CVD can be induced by EDC exposure through direct or indirect effects, and ceRNAs regulate them to varying degrees. When the organism is exposed to EDCs, miRNA, mRNA, lncRNA, and circRNA in ceRNA networks are regulated to different extents, along with the activation of the Nrf2 and p38 MAPK/NF-κB signaling pathways, leading to AS. Additionally, mRNA or protein in ceRNA networks is altered under EDC exposure, activating signaling pathways such as PINK1/Parkin, TGF-β1/LIMK1, and inducing cardiomyopathy. Exposure to EDCs also leads to elevated mRNA levels of ceRNAs, increasing the risk of CVDs. Moreover, ceRNAs may induce vascular disease by participating in increased NO or ROS induced by EDCs. The involvement of ceRNAs in EDC-induced CVD development has been confirmed in population, animal, and cellular studies.

In recent years, advances in bioinformatics have led to the discovery of various functionally important ncRNAs, thus revealing more complex layers of gene regulatory networks. Some ncRNAs contain miRNA-binding sites and serve as miRNA sponges, thereby sequestering miRNAs, alleviating their repression of target genes, and consequently enhancing target gene expression. Reportedly, this interaction forms the basis of extensive ceRNA networks, which allow cells to flexibly adjust gene expression in response to different internal and external environmental stimuli to maintain cellular homeostasis [94].

Based on these findings, constructing and optimising ceRNA regulatory networks could be a viable approach for identifying key targets and pathways. This could advance interventions and treatments for EDC-induced cardiovascular diseases and reduce future mortality rates. However, it is important to note that methodological reporting deficiencies are prevalent in preclinical studies, which poses challenges for our quality assessment. Consequently, we have thoroughly considered these limitations and methodological heterogeneity when synthesising evidence strength. The absence of a unified, fully standardised quantitative scoring system is a potential limitation of this review and reflects a common challenge in the systematic evaluation of preclinical studies. Overall, this study reveals that ceRNA regulatory networks are a highly promising area of research in EDC-induced cardiovascular disease studies. However, research in this area is insufficient and further exploration is needed to identify key target genes and signalling pathways.

Considering the established role of PM2.5 as a major environmental risk factor for CVDs, investigating its potential to dysregulate ceRNA-mediated gene expression may reveal novel molecular mechanisms and significantly broaden the therapeutic landscape. Hence, exploring the potential connection between PM2.5 exposure and ceRNA networks represents a compelling direction for future research. Such studies may provide a foundation for developing novel therapeutic strategies against EDC-mediated cardiovascular damage.

## Abbreviations

Ang II: Angiotensin II
AS: Atherosclerosis
AT1aR: Angiotensin type 1a receptor
ATP: Adenosine triphosphate
A-SMA: A-smooth muscle actin
BM: Basement membrane
BP-3: Benzophenone-3
BPA: Bisphenol A
BPAF: Bisphenol AF
CD163^+^: Cluster of differentiation 163
CD44: Cluster of differentiation 44
CeRNA: Competing endogenous RNAs
CeRNET: CeRNA network
CHD: Coronary heart disease
CI: Complex I
CircRNA: Circular RNAs
CIV: Complex IV
CVD: Cardiovascular disease
DEHP: Diethylhexylphenyl dicarboxylate
DES: Hexestrol
DiNP: Diisononyl phthalate
EC: The human endothelial cell
EDCs: Environmental endocrine disruptors
GPX4: Glutathione peroxidase 4
HF: Heart failure
IL-6: Interleukin-6
IOX: Iodoxynitrile
LncRNA: Long-chain non-coding RNAs
MDA: Malondialdehyde
MiRNA: MicroRNA
MRC: Mitochondrial respiratory complex
MRNA: Messenger ribonucleic acid
NcRNA: Non-coding RNAs
Nkx2.5: NK2 homology box 2.5
NO: Nitric oxide
NP: Nonylphenol
Nrf2: Nuclear factor erythroid 2-related factor 2
OPG: Osteoprotegerin
PAD: Peripheral artery disease
PCB126: Polychlorinated biphenyls 126
PCB29-pQ: 2,3,5-trichloro-6-phenyl-[1,4]-benzene
PCBs: Polychlorinated biphenyls
PFAS: Perfluoroalkyl substances
PFOS: Perfluorooctane sulfonate
PHD: Pulmonary heart disease
POPs: Persistent organic pollutants
RHD: Rheumatic heart disease
ROS: Reactive oxygen species
SOD: Superoxide dismutase
T2DM: Type 2 diabetes mellitus
TBBPA: Tetrabromobisphenol A
TCDD: 2,3,7,8-tetrachlorodibenzo-p-dioxin
TNF-α: Tumor necrosis factor
TNNC1: Troponin-C
VSMCs: Human umbilical vein endothelial cells
WHO: The World Health Organization
ψm: Mitochondrial membrane potential
ProBNP: Pro-B-type Natriuretic Peptide
TGF-β: Transforming growth factor-β
AVP: Arginine vasopressin
IR: Infrared Spectroscopy
MEHP: Phthalic acid mono-2-ethylexyl ester

## Supplementary information

**Additional file 1:** **Table S1.** Literature search strategies.

**Additional file 2:** **Table S2** Quality assessment ofincluded animals studies.

## Declarations

### Availability of data and material

All data are incorporated into the article and its online supplementary material.

### Fundings

This work was supported by the National Natural Science Foundation of China (22266039); the grant from the “Hundred” Level of High-level Innovative Talents in Guizhou Province ([2020]6014).

## References

[1] Adegoke EO, Rahman MS, Park YJ, Kim YJ, Pang MG. Endocrine-Disrupting Chemicals and Infectious Diseases: From Endocrine Disruption to Immunosuppression. Int J Mol Sci. 2021;22(8). doi: 10.3390/ijms22083939. PMC8069594 33920428

[2] Xu Z, Guan C, Cheng Z, Zhou H, Qin W, Feng J, . Research trends and hotspots of circular RNA in cardiovascular disease: A bibliometric analysis. Noncoding RNA Res. 2024;9(3):930–44. doi: 10.1016/j.ncrna.2024.04.002. 38680417 PMC11047193

[3] Dindinger-Hill K, Hu S, Hickman A, Choudry M, Vehawn J, Snyder J, . Association of Baseline Pre-Diagnosis and Post-Diagnosis Obesity and Weight Change with Cardiovascular Risk and Survival Among Nonmetastatic Prostate Cancer Survivors. Clin Genitourin Cancer. 2024;22(3):102057. doi: 10.1016/j.clgc.2024.02.008. 38503572

[4] Alexiadou K, Hartley A, Tan TM, Khamis R. The cardiovascular effects of GLP-1 receptor agonists beyond obesity and type 2 diabetes: An anti-atherosclerotic action. Trends Cardiovasc Med. 2024;34(8):552–7. doi: 10.1016/j.tcm.2024.03.003. 38555029

[5] Ackerman-Banks CM, Palmsten K, Lipkind HS, Ahrens KA. Association between gestational diabetes and cardiovascular disease within 24 months postpartum. Am J Obstet Gynecol MFM. 2024;6(6):101366. doi: 10.1016/j.ajogmf.2024.101366. 38580094 PMC12228983

[6] Xie Q, Nie M, Zhang F, Shao X, Wang J, Song J, . An unexpected interaction between diabetes and cardiovascular diseases on cognitive function: A cross-sectional study. J Affect Disord. 2024;354:688–93. doi: 10.1016/j.jad.2024.03.040. 38521139

[7] Patel SM, Kang YM, Im K, Neuen BL, Anker SD, Bhatt DL, . Sodium-Glucose Cotransporter-2 Inhibitors and Major Adverse Cardiovascular Outcomes: A SMART-C Collaborative Meta-Analysis. Circulation. 2024;149(23):1789–801. doi: 10.1161/circulationaha.124.069568. 38583093 PMC11149938

[8] Rios FJ, Montezano AC, Camargo LL, Touyz RM. Impact of Environmental Factors on Hypertension and Associated Cardiovascular Disease. Can J Cardiol. 2023;39(9):1229–43. doi: 10.1016/j.cjca.2023.07.002. 37422258

[9] Tian Y, Wu Y, Qi M, Song L, Chen B, Wang C, . Associations of remnant cholesterol with cardiovascular and cancer mortality in a nationwide cohort. Sci Bull (Beijing). 2024;69(4):526–34. doi: 10.1016/j.scib.2023.12.035. 38155000

[10] Liu J, Zhu R, Song J, Sohaib M, Wang S, Mao J. . Limosilactobacillus reuteri consumption significantly reduces the total cholesterol concentration without affecting other cardiovascular disease risk factors in adults: A systematic review and meta-analysis. Nutr Res. 2023;117:1–14. doi: 10.1016/j.nutres.2023.06.004. 37419064

[11] Khan Minhas AM, Sedhom R, Jea ED, Shapiro MD, Panza JA, Alam M, . Global burden of cardiovascular disease attributable to smoking, 1990–2019: ananalysis of the 2019 Global Burden of Disease Study. Eur J Prev Cardiol. 2024;31(9):1123–31. doi: 10.1093/eurjpc/zwae040. 38589018

[12] Kobayashi Y, Yamagishi K, Muraki I, Kokubo Y, Saito I, Yatsuya H, . Corrigendum to “Second hand smoke and the risk of incident cardiovascular disease among never-smoking women” [Preventive Medicine 162 (2022)107145]. Prev Med. 2023;167:107396. doi: 10.1016/j.ypmed.2022.107396. 36581521

[13] Padhi BK, Singh S, Gaidhane AM, Abu Serhan H, Khatib MN, Zahiruddin QS, . Inequalities in cardiovascular disease among elderly Indians: A gender perspective analysis using LASI wave-I (2017–18). Curr Probl Cardiol. 2024;49(7):102605. doi: 10.1016/j.cpcardiol.2024.102605. 38692448

[14] Kapoulea EA, Ready RE, Ginn JC. Loneliness and risk for cardiovascular disease in the United States and Japan: The effects of nationality, collectivism, and gender. Soc Sci Med. 2023;337:116299. doi: 10.1016/j.socscimed.2023.116299. 37837950

[15] Lu Y, Wang R, Norman J, Yu P. Loneliness status transitions and risk of cardiovascular disease among middle-aged and older adults. Nutr Metab Cardiovasc Dis. 2024;34(3):718–25. doi: 10.1016/j.numecd.2023.10.024. 38161117

[16] Smith RT, Olsen TW, Chong V, Kim J, Hammer M, Lema G, . Subretinal drusenoid deposits, age-related macular degeneration, and cardiovascular disease. Asia Pac J Ophthalmol (Phila). 2024;13(1):100036. doi: 10.1016/j.apjo.2024.100036. 38244930

[17] Yilmaz B, Terekeci H, Sandal S, Kelestimur F. Endocrine disrupting chemicals: exposure, effects on human health, mechanism of action, models for testing and strategies for prevention. Rev Endocr Metab Disord. 2020;21(1):127–47. doi: 10.1007/s11154-019-09521-z. 31792807

[18] Chen Y, Yang J, Yao B, Zhi D, Luo L, Zhou Y. Endocrine disrupting chemicals in the environment: Environmental sources, biological effects, remediation techniques, and perspective. Environ Pollut. 2022;310:119918. doi: 10.1016/j.envpol.2022.119918. 35952990

[19] Mentsiou Nikolaou E, Kalafati IP, Dedoussis GV. The Interplay between Endocrine-Disrupting Chemicals and the Epigenome towards Metabolic Dysfunction-Associated Steatotic Liver Disease: A Comprehensive Review. Nutrients. 2024;16(8):1124. doi: 10.3390/nu16081124. 38674815 PMC11054068

[20] Uldbjerg CS, Koch T, Lim YH, Gregersen LS, Olesen CS, Andersson AM, . Prenatal and postnatal exposures to endocrine disrupting chemicals and timing of pubertal onset in girls and boys: a systematic review and meta-analysis. Hum Reprod Update. 2022;28(5):687–716. doi: 10.1093/humupd/dmac013. 35466359 PMC9434240

[21] Khushboo M, Sanjeev S, Murthy MK, Sunitadevi M, Dinata R, Bhanushree B, . Dietary phytoestrogen diosgenin interrupts metabolism, physiology, and reproduction of Swiss albino mice: Possible mode of action as an emerging environmental contaminant, endocrine disruptor and reproductive toxicant. Food Chem Toxicol. 2023;176:113798. doi: 10.1016/j.fct.2023.113798. 37146712

[22] Wang F. Reproductive endocrine disruption effect and mechanism in male zebrafish after life cycle exposure to environmental relevant triclosan. Aquat Toxicol. 2024;270:106899. doi: 10.1016/j.aquatox.2024.106899. 38492288

[23] Lin H, Zhou L, Lu S, Yang H, Li Y, Yang X. Occurrence and spatiotemporal distribution of natural and synthetic steroid hormones in soil, water, and sediment systems in suburban agricultural area of Guang zhou City, China. J Hazard Mater. 2024;470:134288. doi: 10.1016/j.jhazmat.2024.134288. 38626685

[24] Lin HW, Feng HX, Chen L, Yuan XJ, Tan Z. Maternal exposure to environmental endocrine disruptors during pregnancy is associated with pediatric germ cell tumors. Nagoya J Med Sci. 2020;82(2):323–33. doi: 10.18999/nagjms.82.2.315. 32581411 PMC7276419

[25] Hassan S, Thacharodi A, Priya A, Meenatchi R, Hegde TA, R T, . Endocrine disruptors: Unravelling the link between chemical exposure and Women’s reproductive health. Environ Res. 2024;241:117385. doi: 10.1016/j.envres.2023.117385. 37838203

[26] Mohanty B. Pesticides exposure and compromised fitness in wild birds: Focusing on the reproductive endocrine disruption. Pesticide Biochem Physiol. 2024;199:105800. doi: 10.1016/j.pestbp.2024.105800. 38458691

[27] Denic-Roberts H, McAdam J, Sjodin A, Davis M, Jones R, Ward MH, . Endocrine disrupting chemical mixture exposure and risk of papillary thyroid cancer in U.S. military personnel: A nested case-control study. Sci Total Environ. 2024;922:171342. doi: 10.1016/j.scitotenv.2024.171342. 38428594 PMC11034764

[28] Conde-Díaz A, Santana-Mayor Á, Herrera-Herrera AV, Socas-Rodríguez B, Rodríguez-Delgado M. Assessment of endocrine disruptor pollutants and their metabolites in environmental water samples using a sustainable natural deep eutectic solvent-based analytical methodology. Chemosphere. 2023;338:139480. doi: 10.1016/j.chemosphere.2023.139480. 37453517

[29] Zhan W, Qiu W, Ao Y, Zhou W, Sun Y, Zhao H, . Environmental Exposure to Emerging Alternatives of Per- and Polyfluoroalkyl Substances and Polycystic Ovarian Syndrome in Women Diagnosed with Infertility: A Mixture Analysis. Environ Health Perspect. 2023;131(5):57001. doi: 10.1289/ehp11814. 37134253 PMC10156134

[30] Wu L, Gu J, Duan X, Ge F, Ye H, Kong L, . Insight into the mechanisms of neuroendocrine toxicity induced by 6:2FTCA via thyroid hormone disruption. Chemosphere. 2023;341:140031. doi: 10.1016/j.chemosphere.2023.140031. 37660785

[31] Okeke ES, Feng W, Mao G, Chen Y, Qian X, Luo M, . A transcriptomic-based analysis predicts the neuroendocrine disrupting effect on adult male and female zebrafish (Danio rerio) following long-term exposure to tetrabromobisphenol A bis(2-hydroxyethyl) ether. Comp Biochem Physiol C Toxicol Pharmacol. 2023;264:109527. doi: 10.1016/j.cbpc.2022.109527. 36442598

[32] Li Y, Reivan Ortiz GG, Uyen PTM, Cong PT, Othman SI, Allam AA, et al. Environmental impact of endocrine-disrupting chemicals and heavy metals in biological samples of petrochemical industry workers with perspective management. Environ Res. 2023;231(Pt2):115913. doi: 10.1016/j.envres.2023.115913. 37178754

[33] Cheng B, Li C, Li J, Gong L, Liang P, Chen Y, . The activity and immune dynamics of PD-1 inhibition on high-risk pulmonary ground glass opacity lesions: insights from a single-arm, phase II trial. Signal Transduct Target Ther. 2024;9(1):93. doi: 10.1038/s41392-024-01799-z. 38637495 PMC11026465

[34] Cheng IS, Tsao JP, Bernard JR, Tsai TW, Chang CC, Liao SF. Oral post-exercise garlic extract supplementation enhances glycogen replenishment but does not up-regulate mitochondria biogenesis mRNA expression in human-exercised skeletal muscle. J Int Soc Sports Nutr. 2024;21(1):2336095. doi: 10.1080/15502783.2024.2336095. 38576169 PMC11000618

[35] Chen S, Nie H, Huo Z, Yan X. Comprehensive analysis of differentially expressed mRNA, lncRNA and miRNA, and their ceRNA networks in the regulation of shell color in the Manila clam (Ruditapes philippinarum). Int J Biol Macromol. 2024;256(Pt2):128404. doi: 10.1016/j.ijbiomac.2023.128404. 38016607

[36] Su L, Li R, Zhang Z, Liu J, Du J, Wei H. Identification of altered exosomal microRNAs and mRNAs in Alzheimer’s disease. Ageing Res Rev. 2022;73:101497. doi: 10.1016/j.arr.2021.101497. 34710587

[37] Zhang J, Luo Q, Li X, Guo J, Zhu Q, Lu X, . Novel role of immune-related non-coding RNAs as potential biomarkers regulating tumour immunoresponse via MICA/NKG2D pathway. Biomark Res. 2023;11(1):86. doi: 10.1186/s40364-023-00530-4. 37784183 PMC10546648

[38] Di Fusco SA, Aquilani S, Spinelli A, Alonzo A, Matteucci A, Castello L, . The polypill strategy in cardiovascular disease prevention: It’s time for its implementation. Prog Cardiovasc Dis. 2023;79:37–40. doi: 10.1016/j.pcad.2023.03.003. 36931543

[39] Liu J, Hernandez R, Li X, Meng Z, Chen H, Zhou C. Pregnane X Receptor Mediates Atherosclerosis Induced by Dicyclohexyl Phthalate in LDL Receptor-Deficient Mice. Cells. 2022;11(7). doi: 10.3390/cells11071125. PMC8997706 35406689

[40] Shan Q, Qu F, Chen N. 2,3,7,8-Tetrachlorodibenzo-p-dioxin (TCDD) and Polychlorinated Biphenyl Coexposure Alters the Expression Profile of MicroRNAs in the Liver Associated with Atherosclerosis. BioMed Res Int. 2020;2020:2652756. doi: 10.1155/2020/2652756. 32855961 PMC7443005

[41] Mo ZW, Peng YM, Zhang YX, Li Y, Kang BA, Chen YT, . High-density lipoprotein regulates angiogenesis by long non-coding RNA HDRACA. Signal Transduct Target Ther. 2023;8(1):299. doi: 10.1038/s41392-023-01558-6. 37574469 PMC10423722

[42] Liu C, Qin Q, Xu J, Li X, Cong H. Phthalate promotes atherosclerosis through interacting with long-non coding RNA and induces macrophage foam cell formation and vascular smooth muscle damage. Chemosphere. 2022;308(Pt 2):136383. doi: 10.1016/j.chemosphere.2022.136383. 36088979

[43] Fittipaldi S, Bimonte VM, Soricelli A, Aversa A, Lenzi A, Greco EA, . Cadmium exposure alters steroid receptors and proinflammatory cytokine levels in endothelial cells in vitro: a potential mechanism of endocrine disruptor atherogenic effect. J Endocrinol Invest. 2019;42(6):727–39. doi: 10.1007/s40618-018-0982-1. 30478740

[44] Yang B, Ye Z, Wang Y, Guo H, Lehmler HJ, Huang R, . Evaluation of Early Biomarkers of Atherosclerosis Associated with Polychlorinated Biphenyl Exposure: An in Vitro and in Vivo Study. Environ Health Perspect. 2022;130(3):37011. doi: 10.1289/ehp9833. 35349355 PMC8963524

[45] Liu J, Yang B, Wang Y, Wu Y, Fan B, Zhu S, . Polychlorinated biphenyl quinone promotes macrophage polarization to CD163(+) cells through Nrf2 signaling pathway. Environ Pollut. 2020;257:113587. doi: 10.1016/j.envpol.2019.113587. 31801669

[46] Yang B, Qin Q, Xu L, Lv X, Liu Z, Song E, . Polychlorinated Biphenyl Quinone Promotes Atherosclerosis through Lipid Accumulation and Endoplasmic Reticulum Stress via CD36. Chem Res Toxicol. 2020;33(6):1497–507. doi: 10.1021/acs.chemrestox.0c00123. 32434321

[47] Gu J, Wang H, Zhou L, Fan D, Shi L, Ji G, . Oxidative stress in bisphenol AF-induced cardiotoxicity in zebrafish and the protective role of N-acetyl N-cysteine. Sci Total Environ. 2020;731:139190. doi: 10.1016/j.scitotenv.2020.139190. 32408210

[48] Li YF, Rodrigues J, Campinho MA. Ioxynil and diethylstilbestrol increase the risks of cardiovascular and thyroid dysfunction in zebrafish. Sci Total Environ. 2022;838(Pt 3):156386. doi: 10.1016/j.scitotenv.2022.156386. 35662599

[49] Lamberto F, Shashikadze B, Elkhateib R, Lombardo SD, Horánszky A, Balogh A, . Low-dose Bisphenol A exposure alters the functionality and cellular environment in a human cardiomyocyte model. Environ Pollut. 2023;335:122359. doi: 10.1016/j.envpol.2023.122359. 37567409

[50] Gear R, Kendziorski JA, Belcher SM. Effects of bisphenol A on incidence and severity of cardiac lesions in the NCTR-Sprague-Dawley rat: A CLARITY-BPA study. Toxicol Lett. 2017;275:123–35. doi: 10.1016/j.toxlet.2017.05.011. 28499613 PMC5526598

[51] Ma J, Ross L, Grube C, Wang HS. Toxicity of low dose bisphenols in human iPSC-derived cardiomyocytes and human cardiac organoids - Impact on contractile function and hypertrophy. Chemosphere. 2024;353:141567. doi: 10.1016/j.chemosphere.2024.141567. 38417488

[52] Koneva LA, Vyas AK, McEachin RC, Puttabyatappa M, Wang HS, Sartor MA, . Developmental programming: Interaction between prenatal BPA and postnatal overfeeding on cardiac tissue gene expression in female sheep. Environ Mol Mutagen. 2017;58(1):4–18. doi: 10.1002/em.22071. 28079927 PMC5730970

[53] Jiang Y, Xia W, Yang J, Zhu Y, Chang H, Liu J, . BPA-induced DNA hypermethylation of the master mitochondrial gene PGC-1α contributes to cardiomyopathy in male rats. Toxicology. 2015;329:21–31. doi: 10.1016/j.tox.2015.01.001. 25572651

[54] Yujiao C, Meng Z, Shanshan L, Wei W, Yipeng W, Chenghong Y. Exposure to Bisphenol A induces abnormal fetal heart development by promoting ferroptosis. Ecotoxicol Environ Saf. 2023;255:114753. doi: 10.1016/j.ecoenv.2023.114753. 36933485

[55] Zhang Y, Xu J, Liu C, Long X, Zheng M, He J, . Curative effect of zinc-selenium tea on rat’s cardiotoxicity induced by long-term exposure to nonylphenol. Environ Toxicol. 2023;38(1):101–14. doi: 10.1002/tox.23667. 36239032

[56] Guo M, Xu J, Long X, Liu W, Aris AZ, Yang D, . Myocardial fibrosis induced by nonylphenol and its regulatory effect on the TGF-β1/LIMK1 signaling pathway. Ecotoxicol Environ Saf. 2024;272:116110. doi: 10.1016/j.ecoenv.2024.116110. 38364763

[57] Ni C, Pan K, Xu J, Long X, Lin F, Nie Y, . Effects and mechanism of perinatal nonylphenol exposure on cardiac function and myocardial mitochondria in neonatal rats. Ecotoxicol Environ Saf. 2023;258:114977. doi: 10.1016/j.ecoenv.2023.114977. 37146387

[58] Yang X, Xu J, Xu Y, Wang C, Lin F, Yu J. Regulatory mechanism of perinatal nonylphenol exposure on cardiac mitochondrial autophagy and the PINK1/Parkin signaling pathway in male offspring rats. Phytomedicine. 2024;126:155434. doi: 10.1016/j.phymed.2024.155434. 38367424

[59] Ortiz-Villanueva E, Jaumot J, Martínez R, Navarro-Martín L, Piña B, Tauler R. Assessment of endocrine disruptors effects on zebrafish (Danio rerio) embryos by untargeted LC-HRMS metabolomic analysis. Sci Total Environ. 2018;635:156–66. doi: 10.1016/j.scitotenv.2018.03.369. 29660719

[60] Gan M, Zheng T, Shen L, Tan Y, Fan Y, Shuai S, . Genistein reverses isoproterenol-induced cardiac hypertrophy by regulating miR-451/TIMP2. Biomed Pharmacother. 2019;112:108618. doi: 10.1016/j.biopha.2019.108618. 30798118

[61] Lai KP, Gong Z, Tse WKF. Zebrafish as the toxicant screening model: Transgenic and omics approaches. Aquat Toxicol. 2021;234:105813. doi: 10.1016/j.aquatox.2021.105813. 33812311

[62] Rehman A, Kumari R, Kamthan A, Tiwari R, Srivastava RK, van der Westhuizen FH, . Cell-free circulating mitochondrial DNA: An emerging biomarker for airborne particulate matter associated with cardiovascular diseases. Free Radic Biol Med. 2023;195:103–20. doi: 10.1016/j.freeradbiomed.2022.12.083. 36584454

[63] Kehinde SA, Ore A, Olajide AT, Ajagunna IE, Oloyede FA, Faniyi TO, . Diisononyl phthalate inhibits cardiac glycolysis and oxidative phosphorylation by down-regulating cytosolic and mitochondrial energy metabolizing enzymes in murine model. Adv Redox Res. 2022;6:100041. doi: 10.1016/j.arres.2022.100041.

[64] Li YF, Rodrigues J, Campinho MA. Ioxynil and diethylstilbestrol increase the risks of cardiovascular and thyroid dysfunction in zebrafish. Sci Total Environ. 2022;838(Pt3):156386. doi: 10.1016/j.scitotenv.2022.156386. 35662599

[65] Brulport A, Le Corre L, Chagnon MC. Chronic exposure of 2,3,7,8-tetrachlorodibenzo-p-dioxin (TCDD) induces an obesogenic effect in C57BL/6J mice fed a high fat diet. Toxicology. 2017;390:43–52. doi: 10.1016/j.tox.2017.07.017. 28774668

[66] Easson S, Singh RD, Connors L, Scheidl T, Baker L, Jadli A, . Exploring oxidative stress and endothelial dysfunction as a mechanism linking bisphenol S exposure to vascular disease in human umbilical vein endothelial cells and a mouse model of postnatal exposure. Environ Int. 2022;170:107603. doi: 10.1016/j.envint.2022.107603. 36335898

[67] Ansari MI, Bano N, Kainat KM, Singh VK, Sharma PK. Bisphenol A exposure induces metastatic aggression in low metastatic MCF-7 cells via PGC-1α mediated mitochondrial biogenesis and epithelial-mesenchymal plasticity. Life Sci. 2022;302:120649. doi: 10.1016/j.lfs.2022.120649. 35597549

[68] Deng P, Wang C, Wahlang B, Sexton T, Morris AJ, Hennig B. Co-exposure to PCB126 and PFOS increases biomarkers associated with cardiovascular disease risk and liver injury in mice. Toxicol Appl Pharmacol. 2020;409:115301. doi: 10.1016/j.taap.2020.115301. 33096110 PMC7725976

[69] Arsenescu V, Arsenescu R, Parulkar M, Karounos M, Zhang X, Baker N, . Polychlorinated biphenyl 77 augments angiotensin II-induced atherosclerosis and abdominal aortic aneurysms in male apolipoprotein E deficient mice. Toxicol Appl Pharmacol. 2011;257(1):148–54. doi: 10.1016/j.taap.2011.08.028. 21925196 PMC3220787

[70] Varra FN, Varras M, Varra VK, Theodosis-Nobelos P. Molecular and pathophysiological relationship between obesity and chronic inflammation in the manifestation of metabolic dysfunctions and their inflammation-mediating treatment options (Review). Mol Med Rep. 2024;29(6). doi: 10.3892/mmr.2024.13219. PMC11025031 38606791

[71] Alshaya OA, Korayem GB, Alghwainm M, Alyami W, Alotaibi A, Alyami MS, . The prevalence of cardiovascular diseases, chronic kidney disease, and obesity in patients with type 2 diabetes mellitus and the description of concurrent treatments: A two-center retrospective cross-sectional study in Saudi Arabia. Saudi Pharm J. 2024;32(5):102054. doi: 10.1016/j.jsps.2024.102054. 38590611 PMC10999870

[72] Soundararajan A, Prabu P, Mohan V, Gibert Y, Balasubramanyam M. Novel insights of elevated systemic levels of bisphenol-A (BPA) linked to poor glycemic control, accelerated cellular senescence and insulin resistance in patients with type 2 diabetes. Mol Cell Biochem. 2019;458(1–2):171–83. doi: 10.1007/s11010-019-03540-9. 31004310

[73] Wu L, Shi F, Zhang Y, Xu X, Xie Z, Hua S, . Maternal exposure to dibutyl phthalate (DBP) impairs angiogenesis and AR signalling pathway through suppression of TGFB1I1 in hypospadias offspring. Ecotoxicol Environ Saf. 2024;270:115941. doi: 10.1016/j.ecoenv.2024.115941. 38184977

[74] Kokai D, Stanic B, Tesic B, Samardzija Nenadov D, Pogrmic-Majkic K, Fa Nedeljkovic S, . Dibutyl phthalate promotes angiogenesis in EA.hy926 cells through estrogen receptor-dependent activation of ERK1/2, PI3K-Akt, and NO signaling pathways. Chem Biol Interact. 2022;366:110174. doi: 10.1016/j.cbi.2022.110174. 36089060

[75] Alvarez-Gonzalez MY, Sánchez-Islas E, Mucio-Ramirez S, de Gortari P, Amaya MI, Kodavanti PRS, . Perinatal exposure to octabromodiphenyl ether mixture, DE-79, alters the vasopressinergic system in adult rats. Toxicol Appl Pharmacol. 2020;391:114914. doi: 10.1016/j.taap.2020.114914. 32032643 PMC8103815

[76] Nehru M, Subramaniam P, Jancy MS, Durairaj P, Kumar JS, Prabhu V. Impact of bisphenol a on the levels of vascular calcification biomarkers in type 2 diabetes mellitus with vascular complications: A case-control study. Emerging Contaminants. 2024;10(4):100342. doi: 10.1016/j.emcon.2024.100342.

[77] Okeke ES, Ezeorba TPC, Chen Y, Mao G, Feng W, Wu X. Association of tetrabromobisphenol A (TBBPA) with micro/nano-plastics: A review of recent findings on ecotoxicological and health impacts. Sci Total Environ. 2024;927:172308. doi: 10.1016/j.scitotenv.2024.172308. 38599396

[78] Song S, Li Y, Lv L, Dong M, Qin Z. Tetrabromobisphenol A exerts thyroid disrupting effects but has little overt impact on postnatal brain development and neurobehaviors in mice. J Environ Sci (China). 2024;142:1–10. doi: 10.1016/j.jes.2023.10.028. 38527875

[79] Feiteiro J, Rocha SM, Mariana M, Maia CJ, Cairrão E. Pathways involved in the human vascular Tetrabromobisphenol A response: Calcium and potassium channels and nitric oxide donors. Toxicology. 2022;470:153158. doi: 10.1016/j.tox.2022.153158. 35321852

[80] Nie Y, Liu H, Wu R, Fan J, Yang Y, Zhao W, . Interference with SPARC inhibits Benzophenone-3 induced ferroptosis in osteoarthritis: Evidence from bioinformatics analyses and biological experimentation. Ecotoxicol Environ Saf. 2024;274:116217. doi: 10.1016/j.ecoenv.2024.116217. 38489904

[81] Morozova E, Kariagina A, Busch C, Schwartz RC. Benzophenone-3 alters expression of genes encoding vascularization and epithelial-mesenchymal transition functions during Trp53-null mammary tumorigenesis. Food Chem Toxicol. 2024;186:114540. doi: 10.1016/j.fct.2024.114540. 38387520 PMC10978255

[82] Tavolari S, Bucci L, Tomasi V, Guarnieri T. Selected polychlorobiphenyls congeners bind to estrogen receptor alpha in human umbilical vascular endothelial (HUVE) cells modulating angiogenesis. Toxicology. 2006;218(1):67–74. doi: 10.1016/j.tox.2005.10.008. 16293362

[83] Wu X, Sui Z, Zhang H, Wang Y, Yu Z. Integrated Analysis of lncRNA-Mediated ceRNA Network in Lung Adenocarcinoma. Front Oncol. 2020;10:554759. doi: 10.3389/fonc.2020.554759. 33042838 PMC7523091

[84] Mariana M, Soares A, Castelo-Branco M, Cairrao E. Exposure to DEP Modifies the Human Umbilical Artery Vascular Resistance Contributing to Hypertension in Pregnancy. J Xenobiot. 2024;14(2):497–515. doi: 10.3390/jox14020030. 38651380 PMC11036297

[85] Wu Y, Wang J, Zhao T, Wei Y, Han L, Shen L, . LncRNAs activate longevity regulation pathway due to aging of Leydig cells caused by DEHP exposure: A transcriptome-based study. Ecotoxicol Environ Saf. 2021;209:111798. doi: 10.1016/j.ecoenv.2020.111798. 33360214

[86] Huang RG, Li XB, Wang YY, Wu H, Li KD, Jin X, . Endocrine-disrupting chemicals and autoimmune diseases. Environ Res. 2023;231(Pt2):116222. doi: 10.1016/j.envres.2023.116222. 37224951

[87] Izic B, Husejnovic MS, Caluk S, Fejzic H, Kundalic BS, Custovic A. Urban Air Pollution Associated with the Incidence of Autoimmune Thyroid Diseases. Med Arch. 2022;76(2):115–21. doi: 10.5455/medarh.2022.76.115-121. 35774048 PMC9233456

[88] J C. Thotakura B, M SK, C SJ. Effects of Endocrine Disrupting Chemicals (EDCs) on Skeletal System Development: A Review. Cureus. 2023;15(9):e46109. doi: 10.7759/cureus.46109. 37900387 PMC10612124

[89] Mínguez-Alarcón L, Gaskins AJ, Meeker JD, Braun JM, Chavarro JE. Endocrine-disrupting chemicals and male reproductive health. Fertil Steril. 2023;120(6):1138–49. doi: 10.1016/j.fertnstert.2023.10.008. 37827483 PMC10841502

[90] Ceylan T, Akin AT, Karabulut D, Tan FC, Taşkiran M, Yakan B. Therapeutic effect of thymoquinone on brain damage caused by nonylphenol exposure in rats. J Biochem Mol Toxicol. 2023;37(11):e23471. doi: 10.1002/jbt.23471. 37466128

[91] Mérida DM, Moreno-Franco B, Marquès M, León-Latre M, Laclaustra M, Guallar-Castillón P. Phthalate exposure and the metabolic syndrome: A systematic review and meta-analysis. Environ Pollut. 2023;333:121957. doi: 10.1016/j.envpol.2023.121957. 37328121

[92] Li W, Guo L, Fang J, Zhao L, Song S, Fang T, . Phthalates and phthalate metabolites in urine from Tianjin and implications for platelet mitochondrial DNA methylation. Front Public Health. 2023;11:1108555. doi: 10.3389/fpubh.2023.1108555. 37181721 PMC10169620

[93] Mariana M, Castelo-Branco M, Soares AM, Cairrao E. Phthalates’ exposure leads to an increasing concern on cardiovascular health. J Hazard Mater. 2023;457:131680. doi: 10.1016/j.jhazmat.2023.131680. 37269565

[94] Yang S, Wang X, Zhou X, Hou L, Wu J, Zhang W, . ncRNA-mediated ceRNA regulatory network: Transcriptomic insights into breast cancer progression and treatment strategies. Biomed Pharmacother. 2023;162:114698. doi: 10.1016/j.biopha.2023.114698. 37060661

[95] Zhang B, Li Q, Lu Y, Wang W, Tian M, Guo J, . KCNQ1OT1/miR-140-5p/PTP4A3 axis is involved in endosulfan-induced vascular endothelial cell migration linking to atherosclerosis. Toxicol Lett. 2025;412:44–54. doi: 10.1016/j.toxlet.2025.07.1415. 40706910

[96] Moawad AM, Awady S, Ali A, Abdelgwad M, Belal S, Taha SHN, . Phthalate Exposure and Coronary Heart Disease: Possible Implications of Oxidative Stress and Altered miRNA Expression. Chem Res Toxicol. 2024;37(5):723–30. doi: 10.1021/acs.chemrestox.3c00423. 38636967

