# Supplementary material for: CeRNA plays a key role in the induction of cardiovascular diseases by environmental endocrine disruptor exposure
Source: Environ Health Prev Med. 2026 Feb 27;31:14. doi: 10.1265/ehpm.25-00165 (PMC12981975; doi:10.1265/ehpm.25-00165)
Supplement: Supplementary file 1 — Additional file 1: Table S1. Literature search strategies. [file ehpm-31-014-s001.docx]

**Supplementary materials**

| **Table S1. Literature search strategies.** | |
| --- | --- |
| Database | Search Strategies |
| Pubmed | Search: (("Environmental Endocrine Disruptors"[Mesh] OR "Endocrine Disruptors"[Mesh] OR "endocrine disruptor "[tiab] OR "endocrine disrupting chemical "[tiab] OR "environmental hormone "[tiab] OR "nonylphenol"[tiab] OR "phthalates"[tiab] OR "Polychlorinated Biphenyls"[Mesh] OR "polychlorinated biphenyl "[tiab] OR "organochlorine pesticide "[tiab] OR "octyl phenol"[tiab] OR "Bisphenol A"[tiab] OR "perfluoroalkyl"[tiab] OR "paraben "[tiab]) ) AND (("Cardiovascular Diseases"[Mesh] OR "Hypertension"[Mesh] OR "Myocardial Infarction"[Mesh] OR "Stroke"[Mesh] OR "Peripheral Arterial Disease"[Mesh] OR "cardiovascular disease "[tiab] OR "circulatory system disease "[tiab] OR "hypertension"[tiab] OR "blood pressure"[tiab] OR "myocardial infarction"[tiab] OR "MI"[tiab] OR "ischemic heart disease"[tiab] OR "IHD"[tiab] OR "coronary heart disease"[tiab] OR "pulmonary heart disease"[tiab] OR "peripheral arterial disease"[tiab] OR "PAD"[tiab] OR "stroke"[tiab] OR "macrovascular disease "[tiab])) Filters: from 1972/1/1 - 2026/1/1 Sort by: Publication Date |
| Web of Science | ALL=((((((((((((TS = "endocrine disruptor") OR (TS = "endocrine disrupting chemical")) OR (TS = "environmental hormone")) OR (TS = nonylphenol)) OR (TS = phthalates)) OR (TS = "polychlorinated biphenyl")) OR (TS = "organochlorine pesticide")) OR (TS = "octyl phenol")) OR (TS = "Bisphenol A")) OR (TS = perfluoroalkyl)) OR (TS = paraben)) AND ((((((((TS = "cardiovascular disease") OR (TS = hypertension)) OR (TS = "blood pressure")) OR (TS = "myocardial infarction")) OR (TS = "ischemic heart disease")) OR (TS = "coronary heart disease")) OR (TS = stroke)) OR (TS = "peripheral arterial disease"))) |
| ScienceDirect | Title, abstract, keywords: ("endocrine disruptor" OR "endocrine disrupting chemical" OR "environmental hormone" OR "EDC" OR "xenoestrogen") AND ("cardiovascular disease" OR "heart disease" OR "circulatory system disease" OR "cerebrovascular disease") |
