# Supplementary material for: CeRNA plays a key role in the induction of cardiovascular diseases by environmental endocrine disruptor exposure
Source: Environ Health Prev Med. 2026 Feb 27;31:14. doi: 10.1265/ehpm.25-00165 (PMC12981975; doi:10.1265/ehpm.25-00165)
Supplement: Supplementary file 2 — Additional file 2: Table S2 Quality assessment ofincluded animals studies. [file ehpm-31-014-s002.doc]

Table S2 Quality assessment ofincluded animals studies

| Quality assessment | Selection bias | | | Performance bias | | Detection bias | |  |  |  |
| --- | --- | --- | --- | --- | --- | --- | --- | --- | --- | --- |
| References | Random sequences generation | Baseline characteristics | Allocation Concealment | Random housing | Blinding | Random outcome assessment | Blinding | Incomeplete outcomes date | Selecting report | Bias from other resources |
| (Shan Q,Qu F,and C.N.2020) | ✓ | × | × | × | × | × | × | ✓ | ✓ | ✓ |
| (Marmugi,A.,et al.,2014) | ✓ | × | × | × | × | ✓ | × | ✓ | ✓ | ✓ |
| (Caroccia,B.,et al.,2023) | ✓ | × | ✓ | ✓ | × | ✓ | × | ✓ | ✓ | ✓ |
| (Jiang,Y.,et al.,2015) | ✓ | × | × | × | ✓ | ? | ✓ | ✓ | ✓ | ✓ |
| (Fittipaldi,S.,et al.,2019) | ✓ | ✓ | ✓ | ? | ? | ? | ? | ? | ? | ? |
| (Brulport,A.,L.LeCorre,and M.C.2017) | ✓ | ✓ | × | × | × | ? | × | ✓ | ✓ | ✓ |
| (Deng,P.,et al.,2020) | ✓ | × | × | × | × | ? | × | ✓ | ✓ | ✓ |
| (Dou,J.,et al.,2023) | ✓ | × | × | × | × | ? | × | ✓ | ✓ | ✓ |
| (Koneva,L.A.,et al.,2017) | ✓ | × | × | × | × |  | × | ✓ | ✓ | ✓ |
| (Kokai,D.,et al.,2022) | ✓ | × | ? | ? | × | ? | × | ✓ | ✓ | ✓ |
| (Ni,C.,et al.,2023) | ✓ | × | × | × | × | ? | × | ? | ✓ | ✓ |
| (Gan,M.,et al.2019) | ✓ | × | × | ? | × | ? | × | ✓ | ✓ | ✓ |
| (Wu,L.,et al.,2024) | ✓ | × | × | ? | × | ? | ✓ | ✓ | ✓ | ✓ |
| (Soundararajan,A.,et al.,2019) | ✓ | ✓ | ? | ✓ | ? | ? | ? | ✓ | ? |  |
| (Gu,J.,et al.,2020) |  |  |  |  |  |  |  |  |  |  |
| (Alvarez-Gonzalez,M.Y.,et al.,2020) | ✓ | × | × | ? | × | ? | × | ✓ | ✓ | ✓ |
| (Liu,C.,et al.,2022) | ✓ | ✓ | ✓ | ? | ? | ? | ? | ? | ✓ | ? |
| (Arsenescu,V.,et al.,2011) | × | ✓ | × | ? | ✓ | ? | ✓ | ✓ | ✓ | ✓ |

The assessment results for each entry are indicated as “Yes,”use “✓”,“No,”use “×”, or “Uncertain,”use “?”, where “Yes” represents low risk bias, ‘No’ represents high risk bias, and “Uncertain” indicates uncertain risk bias.
